# Efficient Degradation of Iopromide by Using Sulfite Activated with Mackinawite

**DOI:** 10.3390/molecules26216527

**Published:** 2021-10-28

**Authors:** Yingtan Yu, Ying Lyu, Ting Zhang, Lin Liu, Bing Fan, Jian Wang, Chaoxing Zhang

**Affiliations:** School of Environment, Liaoning University, Shenyang 110036, China; yuyingtan@lnu.edu.cn (Y.Y.); ly15040420490@163.com (Y.L.); zt18524455863@163.com (T.Z.); linliujx@163.com (L.L.); fandaaa@126.com (B.F.)

**Keywords:** iodinated X-ray contrast medium, FeS, advanced oxidation processes, sulfate radicals

## Abstract

Iopromide (IOP), an iodinated X-ray contrast medium (ICM), is identified as a precursor to iodide disinfection byproducts that have high genotoxicity and cytotoxicity to mammals. ICM remains persistent through typical wastewater treatment processes and even through some hydroxyl radical-based advanced oxidation processes. The development of new technologies to remove ICMs is needed. In this work, mackinawite (FeS)-activated sulfite autoxidation was employed for the degradation of IOP-containing water. The experiment was performed in a 500 mL self-made temperature-controlled reactor with online monitoring pH and dissolved oxygen in the laboratory. The effects of various parameters, such as initial pH values, sulfite dosages, FeS dosages, dissolved oxygen, and inorganic anions on the performance of the treatment process have been investigated. Eighty percent of IOP could be degraded in 15 min with 1 g L^−1^ FeS, 400 μmol L^−1^ sulfite at pH 8, and high efficiency on the removal of total organic carbon (TOC) was achieved, which is 71.8% via a reaction for 1 h. The generated hydroxyl and oxysulfur radicals, which contributed to the oxidation process, were identified through radical quenching experiments. The dissolved oxygen was essential for the degradation of IOP. The presence of Cl^−^ could facilitate IOP degradation, while NO_3_^−^ and CO_3_^2−^ could inhibit the degradation process. The reaction pathway involving H-abstraction and oxidative decarboxylation was proposed, based on product identification. The current system shows good applicability for the degradation of IOP and may help in developing a new approach for the treatment of ICM-containing water.

## 1. Introduction

Iopromide(*N,N*′-bis(2,3-dihydroxypropyl)-2,4,6-triiodo-5-(2-methoxyacetamido)-*N*-methylisophthalamide, IOP), an iodinated X-ray contrast medium (ICM), has been widely used for enabling medical imaging of blood vessels and soft tissues through X-ray examinations [1]. ICMs are metabolically stable in the human body and more than 90% is excreted in the first 24 h through urine or excrement [2]. In previous investigations, ICMs have been detected frequently in the effluent and surface water of sewage treatment plants [3], and even in tap water [4]. In recent years, ICMs have been identified as a precursor to iodide disinfection byproducts (I-DBPs) and have received increasing attention [5]. Studies have shown that ICMs are a major source of iodine during the formation of iodo-trihalomethanes (I-THM) and iodic acid disinfection intermediates (iodo-acids) [6]. These two classes of substances have high genotoxicity and cytotoxicity to mammals [7]. The metabolites or transformation products of ICM in the environment are likely to be detrimental to the environment and human health. The mineralization of ICMs, especially in water treatment systems, is important to avoid any potential harm caused by pernicious byproducts. However, ICMs are recalcitrant to traditional wastewater treatment processes and even in common drinking-water treatment plants [8]. The development of new degradation strategies to remove ICMs is needed.

Advanced oxidation processes (AOPs) have been widely tested for the degradation of ICMs. A variety of systems, including O_3_/H_2_O_2_, UV/TiO_2_, and UV/chloramines systems, have been applied for the degradation of ICMs [9]. However, not all of these can achieve effective degradation. The rate of degradation of IOP (k = 0.18 min^−1^) was observed to be lower than that of other pharmaceutical products in the presence of 0.5 g L^−1^ or 1 g L^−1^ TiO_2_ with a xenon lamp or UV-A light irradiation [10], and the mineralization rate was also modest (<15%) in a laboratory with simulated wastewater. Effective removal was also not achieved via ozonation or O_3_/H_2_O_2_ oxidation [11]. AOPs based on sulfate radicals (SR-AOPs) have shown good efficiency in removing various pollutants [12] and have therefore been used for the oxidation of ICMs, such as iohexol [13] and IOP [14]. It has been discovered that SO_4_^•−^ has a higher redox potential (2.5–3.1 V, normal hydrogen electrode) than HO• (1.9–2.7 V, normal hydrogen electrode) [15]. SO_4_^•−^ exhibits higher selectivity for oxidation than HO• [16]. SO_4_^•−^ are usually generated by activated persulfate (PS) or peroxymonosulfate (PMS) via transition metals [17], heat [18], ultraviolet irradiation [19], alkali [20], and ultrasonication [21]. However, PS and PMS have several drawbacks, such as high cost, inherent toxicity, and residue problems [22]. Recently, the use of activated sulfite to generate SO_4_^•−^ has attracted considerable attention. Sulfite is considered to be a promising alternative to PS or PMS because of its low toxicity and competitive price, where the price of sulfite is about 50% lower than PS and almost 10 times lower than PMS. In general, sulfite could be activated by various transitional metals, including Fe(II/III/VI), Mn(II/III/VII), Co(II), Cu(II), and Cr(III/VI), which involves one-electron transfer from S(IV) to metal ions (M^n+^), thereby generating SO_3_^•−^ [23]. Then, SO_3_^•−^ quickly reacts with oxygen to give SO_5_^•−^, and SO_4_^•−^ could be formed through reduction of SO_5_^•−^ with bisulfite, as shown in reaction (1–8) [24,25].

Among the transition metals used for sulfite catalysis, the use of iron is most widely reported [26] due to its abundance in the environment, high catalysis efficiency, low toxicity, and low cost [27]. Fe(III) and SO_4_^2−^ formed in the reaction are electron acceptors that are also beneficial for the biodegradation of organic pollutants [28]. However, ferrous ion has some drawbacks as catalysts, including its acidic or near neutral circumstances, slow circulation between Fe(II) and Fe(III), and accumulation of iron sludge [29]. The heterogeneous catalysts for the activation of sulfite have attracted intense interest for water treatment applications. Recently, mackinawite (FeS), a tetragonal ferrous sulfide crystal generated through the dissimilatory bacterial reduction of sulfate [30], has attracted increasing attention for use in water treatment. It has been tested as a catalyst for activating PS to efficiently remove 2,4-dichlorophenoxyacetic acid, *p*-chloroaniline [31], trichloroethene, Orange-G, and tetracycline [32], while its applications in activating sulfite are much less. Chen et al. reported a high degradation efficiency (95%) on propranolol by FeS-activated sulfite systems at pH 6 but insufficient mineralization efficiency (less than 20%) [33]. Whether this treatment method can effectively remove IOP and achieve high TOC removal efficiency would be interesting to study. Moreover, the optimal treatment parameters are also very important for enhancing the mineralization, which still needs further studies.

Fe^3+^ + HSO_3_^−^ → FeSO_3_^+^ + H^+^            (log*K*_1_ = 2.45) (1)

FeSO_3_^+^ → Fe^2+^ + SO_3_^•−^          (*k*_2_ = 0.19 s^−1^) (2)

Fe^2+^ + HSO_3_^−^ → FeHSO_3_^+^          (log*k*_3_ = 4) (3)

FeHSO_3_^+^ + 1/4O_2_ → FeSO_3_^+^ + H_2_O          (*k*_4_ = 1.69 × 10^3^ L mol^−1^s^−1^) (4)

   SO_3_^•−^+ O_2_ → SO_5_^•−^          (*k*_5_ = (1 – 2.3) × 10^9^ L mol^−1^ s^−1^) (5)

SO_5_^•−^+ HSO_3_^−^ → SO_3_^•−^+ HSO_5_^−^          (*k*_6_ ≤ 3 × 10^5^ L mol^−1^ s^−1^) (6)

Fe^2+^ + HSO_5_^−^ → SO_4_^•−^+ Fe^3+^ +OH^−^          (*k*_7_ = 10^4^ – 10^7^ L mol^−1^ s^−1^) (7)

SO_5_^•−^ + HSO_3_^−^ → SO_4_^2−^ + SO_4_^•−^ + H^+^          (*k*_8_ = ~1.2 × 10^4^ L mol^−1^ s^−1^) (8)

In this work, the potential of the FeS/sulfite system for the degradation of IOP, selected as a model pollutant, was investigated. The operational parameters, including initial pH values, sulfite dosage, and FeS dosage on the degradation process, were optimized. The reactive species that contributed to the degradation process were identified through a radical and oxygen quenching experiment to reveal the mechanism of IOP degradation. The degradation products were identified and the proposed primary oxidation pathway was estimated. Finally, the influences of inorganic anions (Cl^−^, CO_3_^2−^ and NO_3_^−^) that existed commonly in natural water were also investigated. 

## 2. Results

### 2.1. Characterization of FeS

**Figure 1:**
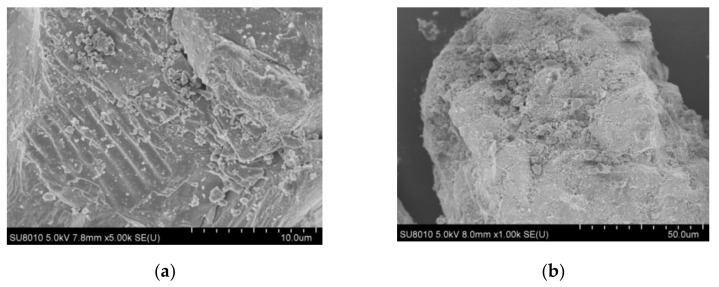
(**a**) SEM images of raw FeS. (**b**) SEM images of the residual FeS.

The morphology of FeS was investigated by using the scanning electron microscope (SEM). As can be clearly seen in Figure 1a,b, the surface of FeS has aggregated micrometer-scale FeS particles. The stacking of these smaller particles causes the FeS surface to exhibit a thin plate-like structure with rugged surfaces and pleated edges [34]. This special sheet-like structure provides a large number of sites for the attachment and reaction of pollutants and free radicals.

The phase structure of FeS was studied by X-ray diffraction (XRD) analysis. The recorded XRD pattern of the FeS particles is shown in Figure S1a. FeS was dovetailed with tetragonal structure planes with space group P4/nmm (129), corresponding to the standard card of FeS (JCPDS No. 14-0117) [35] assigned to the (101), (110), (111), (200), and (201) planes.

Results from the energy dispersive spectrometer (EDS) revealed that the content of S in FeS particles is 34.52%, as shown in Figure S1b. This finding suggests that the S content in the ore primarily corresponds to FeS formation. Therefore, the nominal purity of raw FeS is 50%, and its iron content is 62.44%. Excessive Fe (12.44%) and O (1.39%) contents may have originated from amorphous iron oxide formation [28]. The content of C (4.22%) in the raw FeS is small. The oxidation or degradation reaction was found to usually start from the outside of the FeS particles and proceed inward [36].

Fourier transform infrared spectroscopy (FTIR) was employed to investigate the functional groups, which is shown in Figure S1c ranging from 400 to 4000 cm^−1^. At 412–425 cm^−^^1^, there are very obscure bands that are assigned to Fe(II)-S stretching vibration [37]. The characteristic peaks at around 600 cm^−^^1^ are related to the stretching vibrations of S-S and the Fe-S bond [38]. A significant peak at about 1137 cm^−^^1^ is attributed to the vibration of Fe=S [37]. This peak is diminished observably after the degradation process which reveals that the reaction between FeS and sulfite may destroy the Fe=S bond on the surface of FeS. The peak at 1615 cm^−^^1^ indicates H_2_O bending vibrations [39], while some reports consider this peak is also probably assigned to C=NH [40]. The peak at 3420 cm^−1^ represents the stretching mode of the surface-bonded H_2_O molecules [39], which indicated there still exists water in the sample even after being vacuum dried.

### 2.2. Control Experiments

**Figure 2:**
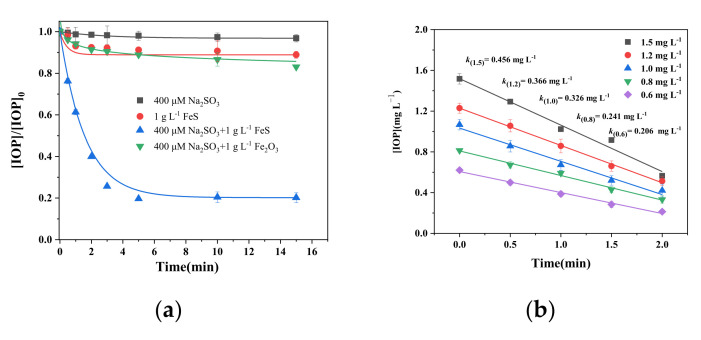

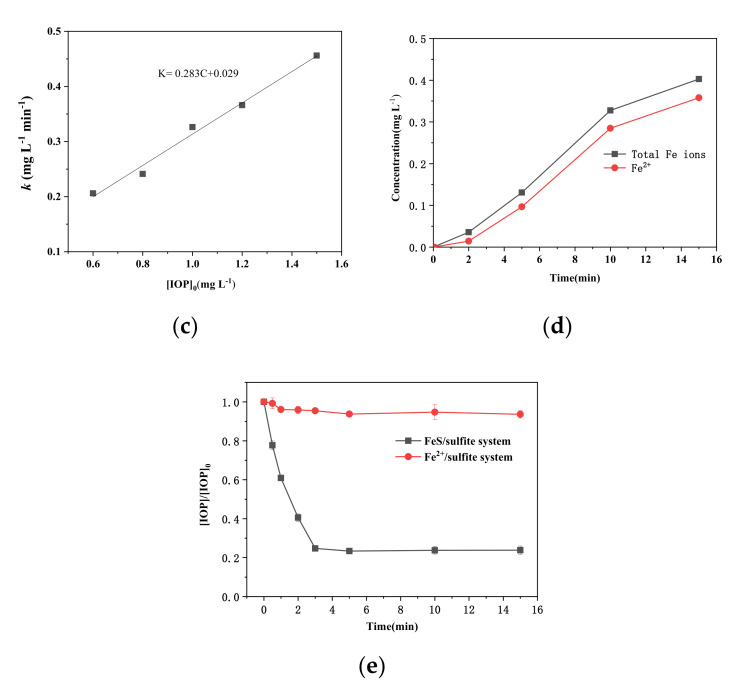
(**a**) Concentration changes of IOP in the control experiments by FeS/sulfite systems. (**b**) The effects of initial IOP concentrations. (**c**) Pseudo-first-order kinetics. (**d**) The concentration changes of total Fe ions and Fe^2+^ in FeS/sulfite system. (**e**) Degradation of IOP by Fe^2+^/sulfite system and FeS/sulfite system. Conditions: [IOP]_0_ = 1.5 mg L^−1^, [FeS]_0_ = [Fe_2_O_3_]_0_ = 1 g L^−1^, Fe^2+^ = 0.4 mg L^−1^ [Na_2_SO_3_]_0_ = 400 μmol L^−1^, pH_init_ = 8.0.

The degradation of IOP via activation of sulfite by FeS is shown in Figure 2a. The results show that in the presence of 400 μmol L^−1^ of sulfite alone, no degradation of IOP occurred in 15 min, while a small amount of about 10% IOP disappeared in the presence of only 1 g L^−1^ of FeS. This slight disappearance may be caused by the reducibility of FeS to reduce various halogenated organic compounds such as trichloroethylene, tetrachloroethylene [41], and *p*-chloroaniline via surface deprotonation or electron transfer, especially in alkaline pH [42]. Eighty percent of IOP could be degraded in 15 min with 1 g L^−1^ of FeS and 400 μmol L^−1^ of sulfite. This degradation was due to the intense oxidizability of SO_4_^•−^ and •OH generated from the autoxidation of sulfite catalyzed by FeS. From the results of EDS, there are small fractions of oxygen that may be from the oxidized surface FeS. In order to exclude the catalysis effect of Fe_2_O_3_, 1 g L^−1^ of Fe_2_O_3_ was employed as the catalyst and the results show that only 14% of IOP was degraded, which can reveal the low effect of Fe_2_O_3_ on the catalysis of sulfite. The reaction of Fe(II)–sulfite in alkaline pH is different from that in acidic or near-neutral pH, which involves the coordination of the Fe(II)–OH complex and SO_3_^2−^ to form the S(IV)–Fe(II)–OH complex [43]. The formed S(IV)–Fe(II)–OH complex could be oxidized into a S(IV)–Fe(III)–OH complex by the dissolved oxygen (DO) in an aqueous solution. SO_3_^•−^ could be released into the aqueous solution with one-electron transfer from Fe(III) to S(IV) of the S(IV)–Fe(III)–OH complex, resulting in the formation of a S(IV)–Fe(II)–OH complex again. SO_3_^•−^ is oxidized into SO_5_^•−^ as the initiation of the oxysulfur species evolves. SO_4_^•−^ and SO_4_^2−^ are generated through the disproportional oxidation of sulfite by SO_5_^•−^ [43]. 

Results in Figure 2a also show a two-stage kinetic curve, including a fast stage in which about 75% of IOP could be degraded in the first 2 min and a slow one thereafter. This curve is a typical degradation kinetics curve that exists for degradation via autoxidation of sulfite, and it was also observed in our previous studies [44]. This curve is considered as being caused by the fast consumption of sulfite and DO [45]. In the fast stage (first 2 min), the concentration of sulfite and DO is relatively high, which leads to the production of a higher amount of the oxysulfur species. Then, DO and sulfite concentrations drop sharply, and the concentrations of the reactive radical species decrease, which retard the degradation of IOP. The initial IOP degradation rate increased with the initial IOP concentration, which followed the pseudo-first-order kinetics shown in Figure 2b,c.

Fe ions could be released from the surface of FeS through reaction (9) [46], which may also form activated sulfite. To confirm this, the concentrations of released Fe ions in the present system were measured using the 1,10-phenanthroline method [47] and the limitation of detection and quantification were determined as 0.022 mg L^−1^ and 0.074 mg L^−1^. The total dissolved Fe ion concentration in 15 min was less than 0.4 ± 0.05 mg L^−1^ (Fe^2+^ concentration was less than 0.36 ± 0.04 mg L^−1^) as shown in Figure 2d. In order to further examine the role of Fe^2+^ in the activation of sulfite, the degradation of IOP by the dissolved Fe^2+^/sulfite system was performed. Results in Figure 2e showed no degradation was found within 15 min. Therefore, FeS/sulfite system is considered to prove heterogeneous activation processes.

FeS + 2O_2_ = Fe^2+^ + SO_4_^2^^−^ (9)

### 2.3. Effects of Initial pH

**Figure 3:**
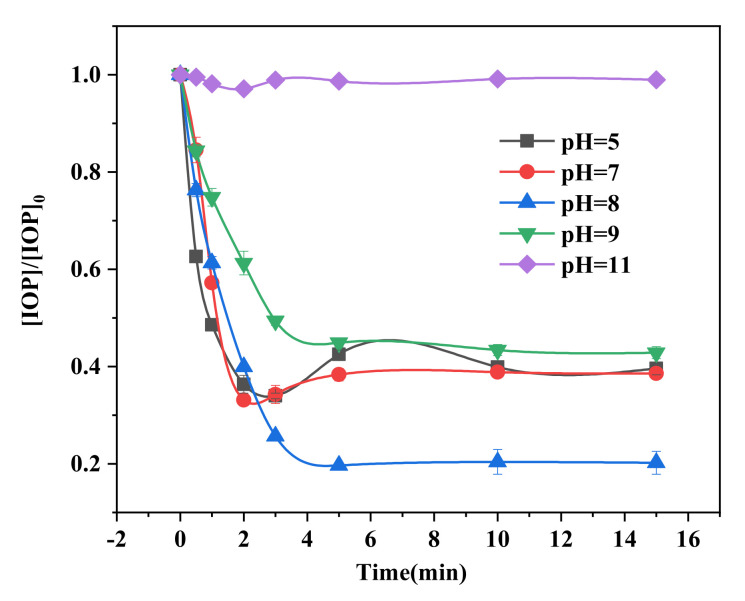
Effects of pH on the degradation of IOP by FeS/sulfite system. Conditions: [IOP]_0_ = 1.5 mg L ^−1^, [FeS]_0_ = 1 g L ^−1^, [Na_2_SO_3_]_0_ = 400 μmo L ^−1^.

pH is a significant parameter in degradation using iron as catalysts. In order to study the effects of the initial pH values, experiments were carried out at five pH values of 5, 7, 8, 9, and 11. The results in Figure 3 indicate that at pH 8, the best performance of degradation of 80% IOP is realized, while there are no significant differences in the degradation efficiency at the other pH values of 5, 7, and 9 (60.4%, 61.4%, and 57.2%, respectively). No degradation could be found at pH 11. The overall effects of pH on the iron-catalyzed autoxidation process of sulfite are rather intricate, where pH can influence the speciation of S(IV) and Fe(II)/Fe(III) and the standard reduction potential of the oxidants. We can explicate the differences in degradation efficiency on the basis of the distribution of S(IV) and Fe(II) at different pH values. The fractions of species and equilibriums shown in Figures S2 and S3 present the distribution of Fe(II) and S(IV) species are both pH-dependent. In alkaline circumstances (pH > 7), the Fe(II) and S(IV) species exist as the ferrous hydroxide complex and SO_3_^2−^ ions, respectively. Then, the S(IV)–Fe(II)–OH complex can be formed, initiating the chain reaction of oxysulfur species in the alkaline solution as mentioned previously. When the pH values increase to 11, iron exists as Fe(OH)_2_(cr), which cannot form a complex with S(IV), resulting in no IOP degradation. On the other hand, in acidic conditions, the bisulfite ion is the predominant species and can form a complex with ferrous ion on the surface of FeS to form FeHSO_3_^+^, and this initiates the auto-oxidation of oxysulfur species that can contribute to the degradation of IOP, as shown in reactions (1–8). However, the excess hydrogen ions can reduce the concentration of sulfite as the precursor of oxysulfur radicals shown in reaction (10), and this can result in a lower IOP-degradation efficiency. Moreover, the pH changes during the degradation process are monitored as shown in Figure S4. Results show, at initial pH values 5 and 7, the pH value declined apace and eventually stay stable at about 3.5, which leads to approximate degradation efficiencies of IOP under weak acid to the neutral condition. While the initial pH is 8, the final pH is 4.4 after the reaction. The pH could affect the conversion of SO_4_^•−^ to HO• through reactions (14) and (15) and result in the different distributions of SO_4_^•−^ and HO•, which could lead to a different degradation efficiency of IOP.

S^2−^ + SO_3_^2−^ + 6H^+^ → 3S + 3H_2_O (10)

### 2.4. Effects of FeS Dosage

**Figure 4:**
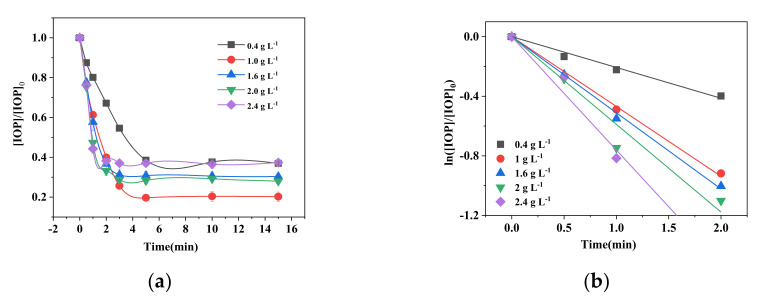
(**a**) Effect of initial FeS dosage on the degradation of IOP by FeS/sulfite system in 15 min. (**b**) Obvious reaction kinetics in first 2 min. Conditions: [IOP]_0_ = 1.5 mg L^−1^, [FeS]_0_ = 0.2–2.4 g L^−1^, [Na_2_SO_3_]_0_ =400 μmol L^−1^, pH_init_ = 8.0.

The effects of the FeS dosage on IOP degradation were investigated next by adding 0.4, 1.0, 1.6, 2.0, and 2.4 g L^−1^ of FeS to 400 μmol L^−1^ sulfite. The results, shown in Figure 4b, indicate that the initial reaction rate in the first 2 min increased with the Fe dose, which confirmed the catalytical effects of Fe(II) on S(IV). However, the total degradation efficiencies of IOP in 15 min were not fitted with that tendency of in first 2 min. As shown in Figure 4a, the efficiency of IOP degradation increased as the FeS dose was raised from 0.4 to 1.0 g L^−1^, and decreased about 10% with the increase in the FeS dose from 1.0 g L^−1^ to 2.0 g L^−1^. Further, at a FeS dose of 2.4 g L^−1^, only 63% of IOP was degraded. This indicates that FeS plays a significant catalytic role, producing SO_4_^•−^, while excess FeS can scavenge SO_4_^•−^, as shown in reaction (11) [48]. This finding is consistent with the reported strong interaction between SO_4_^•−^ and Fe^2+^ ions where excessive Fe(II) acts as a sulfate-free radical scavenger in solution [49].

Fe^2+^ + SO_4_^•^^−^ → Fe^3+^ + SO_4_^2−^          *k* = 4.6 × 10^9^ M^−^^1^ s^−^^1^ (11)

Figure S5 presents the effects of sulfite concentrations on the degradation of IOP. The results show that the IOP degradation efficiency raised from 61.5% to 80% as the initial sulfite concentration was increased from 200 μmol L^−1^ to 400 μmol L^−1^, which could be attributed to increased sulfite content as the precursor of the oxysulfur radicals. However, when the initial concentration of sulfite was changed from 400 μmol L^−1^ to 700 μmol L^−1^, the IOP degradation rate decreased from 73.7% to 54.3%. This is because excessive sulfite will reduce the concentrations of SO_4_^•−^ through reaction (12) [50] and (13) [51], resulting in a low IOP degradation efficiency.

SO_4_^•^^−^ + HSO_3_^−^ → HSO_4_^−^ + SO_3_^•^^−^ (12)

HSO_5_^−^ + HSO_3_^−^ → 2SO_4_^2−^ + H_2_O (13)

### 2.5. Role of Radicals and Dissolved Oxygen

Generally, SO_4_^•−^, SO_5_^•−^, and SO_3_^•−^ are used as the main reactive oxysulfur radicals in the sulfite activation process. HO• is also considered as a reactive radical that contributes to the degradation of IOP, which could be generated by the reactions between SO_4_^•−^/H_2_O or SO_4_^•−^/HO^−^ as shown in reactions (14) and (15) [52]. In order to confirm the contributions of different active species, EtOH and TBA are employed as the scavenger.

SO_4_^•−^ + H_2_O → HSO_4_^−^ + HO•           *k*_18_ = 8.3 M^−^^1^ s^−^^1^ (14)

SO_4_^•^^−^+ OH^−^ → SO_4_^2−^ + HO•           *k*∼6 × 10^7^ M ^−1^ s ^−1^ (15)

**Figure 5:**
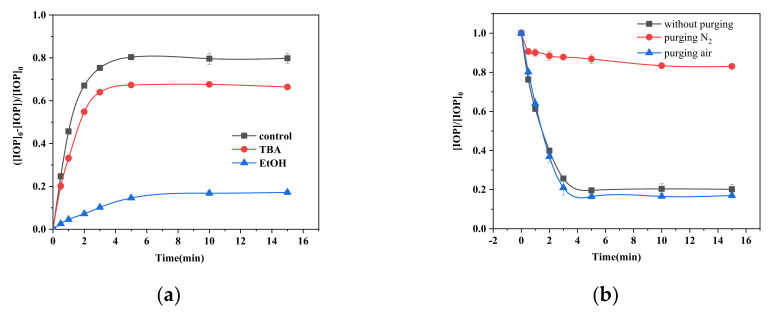
(**a**) Effects of TBA and EtOH on the degradation of IOP by the FeS/sulfite system. (**b**) Effects of purging the FeS/sulfite system with N_2_ or air on IOP degradation. Conditions: [IOP]_0_ = 1.5 mg L ^−1^, [Na_2_SO_3_]_0_ = 400 μmol L ^−1^, [TBA]_0_ = [EtOH]_0_ = 1 mmol L ^−1^, pH_init_ = 8.

EtOH is usually used as an effective scavenger for both SO_4_^•−^ ((1.6–7.7) × 10^7^ M^−1^ s^−1^) and HO• (1.9 × 10^9^ M^−1^ s^−1^) [53]. The rate constant for the reaction between TBA and HO• is (3.8–7.6) × 10^8^ M^−1^ s^−1^, which is 1000 times higher than that for the reaction between TBA and SO_4_^•−^ ((4–9.1) × 10^5^ M^−1^ s^−1^) [16]. A moderate amount of TBA could inhibit HO• alone rather than SO_4_^•−^. As shown in Figure 5a, in the presence of 1 mmol L^−1^ of EtOH, a degradation of only 17.2% IOP occurs, but with no scavenger, the degradation rate is 80%. This means this 17.2% degradation was contributed to the SO_3_^•−^/SO_5_^•−^ since the rate constant between SO_3_^•−^/SO_5_^•−^ and ethanol are very low (k ≤ 10^3^ M^−1^ s^−1^) [54] and 67.6% of IOP were degraded with 1 mmol L^−1^ of TBA indicating that about 50.4% of the degradation was attributed to SO_4_^•−^ and that HO• was responsible for about 12.4% of the degradation.

As shown in reaction (5), the process of generating SO_5_^•−^ by oxidation of SO_3_^•−^ is an important step in the production of SO_4_^•−^ using the present system. DO should be one of the significant factors influencing the degradation of IOP. In order to exclude DO from the present system, the reaction solution was purged with nitrogen to eliminate DO. Figure 5b shows that in the case of nitrogen purging, there was no significant change in the IOP concentration. For 15 min of reaction time, the IOP degradation rate was only 11%, which indicates that the effects from SO_3_^•−^ are weak since SO_5_^•−^ cannot be generated from SO_3_^•−^ without DO. However, when the present system was purged with air, approximately 83% of the IOP was degraded in 15 min. This small enhancement can be attributed to the increased DO concentration leading to the formation of a larger amount of SO_5_^•−^, and subsequently, of a larger amount of SO_4_^•−^.

### 2.6. Effects of Inorganic Anions

The effects of common inorganic anions that exist in natural water including Cl^−^, NO_3_^−^, and CO_3_^2−^ on the degradation of IOP were investigated. As shown in Figure S6a, the IOP degradation efficiency was slightly enhanced with the addition of Cl^−^ (10 and 100 μmol L^−1^). This might be because Cl^•^ could be generated via Cl^−^ competing for the reactive oxidizing species, as shown in reactions (16) and (17). Cl^•^ is selective and can thus degrade some organic pollutants with high rate constants of reaction [53]. However, with raising concentrations of Cl^−^, an increasing amount of SO_4_^•−^ would be consumed. Cl_2_^•−^ and ClHO^−^ with less reactivity might be produced through reactions (18–20), so if the Cl^−^ levels are too high, this might lead to some inhibition of organic-pollutant degradation [31]. This explains why the IOP degradation rate is greater when 10 μmol L^−1^ of chloride ion is added to the reaction system than when 100 μmol L^−1^ is added.

SO_4_^−^ + Cl^−^ → SO_4_^2−^ + Cl^•^            k_19_ = (1.3 ~ 3.1) × 10^8^ M^−^^1^ s^−^^1^ (16)

HO^•^ + Cl^−^ → OH ^−^ + Cl^•^           k_20_ = 4.3× 10^9^ M^−^^1^ s^−^^1^ (17)

  Cl^•^ + Cl ^−^ → Cl_2_^−^           k_21_ = (0.65 ~ 2.1) × 10^10^ M^−^^1^ s^−^^1^ (18)

Cl^•^ + H_2_O → ClHO^−^+ H^+^ (19)

Cl_2_^•^^−^ + H_2_O → ClHO^−^+ H^+^ + Cl ^−^ (20)

Although NO_3_^−^ indicates an inhibiting influence on the degradation of IOP, the effect was very slight, as shown in Figure S6b. This might be because of the adsorption of NO_3_^−^ on the surface of FeS and its reduction [55], thus competing with the active sites for sulfite activation. In Figure S6c, the IOP degradation efficiency decreased with increasing concentration of CO_3_^2−^ from 10 to 100 μmol L ^−1^ in the FeS/sulfite system. For instance, the IOP degradation efficiency was only 56.9% with 100 μmol L^−1^ CO_3_^2−^, but it can achieve approximately 80% in the absence of CO_3_^2^. This result can be reasonably explained by the fact that CO_3_^•−^, which is less reactive than SO_4_^•−^, can be generated through a competitive reaction with SO_4_^•−^ and CO_3_^2-^ [56].

### 2.7. Removal of TOC

**Figure 6:**
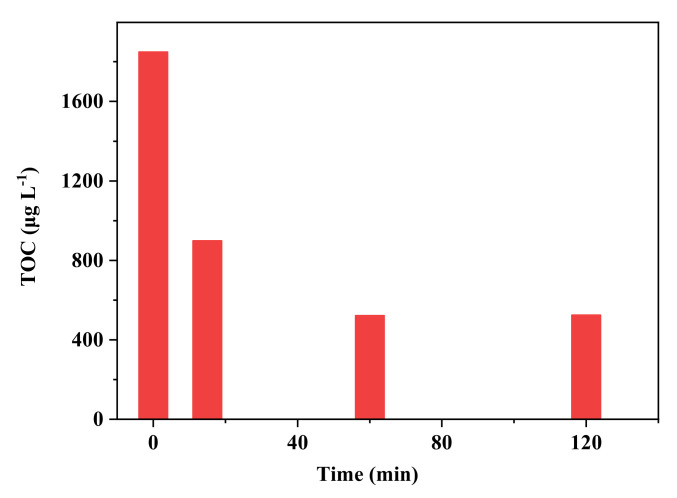
The removal of TOC by FeS/sulfite system. Conditions: [IOP]_0_ = 1.5 mg L^−1^, [FeS]_0_ = 1 g L^−1^, [Na_2_SO_3_]_0_ =400 μmol L ^−1^, pH_init_ = 8.0.

According to the above discussion, the efficiency of mineralization is an important factor for the removal of IOP. In the current system, the removal of TOC was evaluated, which is shown in Figure 6. The TOC reduction achieved 51.4% after 15 min; it then reached 71.8% after 1 h and tended to remain stable thereafter. Chan et al. reported near-complete mineralization of IOP using the UV/peroxydisulfate system after approximately 70–80 min [14], which proves the applicability of SR-AOPs for the mineralization of IOP. 

### 2.8. Determination of Degradation Products

**Figure 7:**
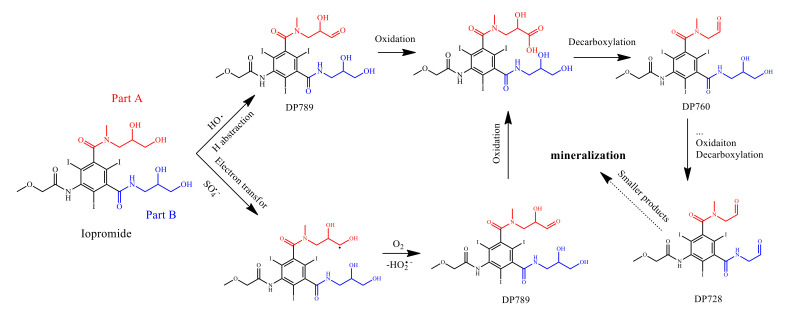
Proposed pathways for IOP degradation in the FeS/sulfite system.

Three primary degradation products (DPs) formed by IOP degradation were identified by LC-MS. All DPs and their fragments, determined from mass spectra, are presented in Table S1 and Figure S7, and these are denoted as DP789 (empirical formula C_18_H_22_I_3_N_3_O_8_), DP760 (empirical formula C_17_H_20_I_3_N_3_O_7_), and DP728 (empirical formula C_16_H_16_O_6_N_3_I_3_) based on their own molecular weight. The fragments of DPs are consistent with those found from photoinduced transformation [57] or electrochemical treatment [5] of IOP. There are two similar glycol structures at the end parts of IOP, namely part A and part B as shown in Figure 7, which are most likely to be oxidized, initiating the degradation of IOP. Further, DP789 is formed by the loss of two hydrogens from IOP, which could be formed by the oxidation of the terminal hydroxyl group to the aldehyde or the oxidation of the central hydroxyl group of the molecular chain to the keto moieties at the molecular chain containing the hydroxyl group at both ends [57]. Regardless of which parts are the first to be oxidized, the products, including DP760 and DP728, indicate that both of the glycol structures are finally oxidized to the aldehyde group and form DP728. The above-mentioned oxidation process could be formed by either HO• or SO_4_^•−^. HO• is preferred to process abstraction of H atoms from the α-H and hydroxyl group, as IOP continually forms aldehyde derivatives or keto moieties derivatives, while SO_4_^•−^ is usually preferred to process electron transformation. However, some organic compounds with less electron-rich groups can also be decomposed by SO_4_^•−^ through the process of H-abstraction [58]. The oxidation process of IOP is very likely initiated through H-abstraction by SO_4_^•−^ since α-carbon is vulnerable to the attack by SO_4_^•−^. The carbon-centered radicals could be formed during the H-abstraction process and could be then rapidly converted to peroxide radicals through oxygen addition. Subsequently, hydroperoxide radicals (HO_2_^•−^) are eliminated from the formed peroxide radicals [59] to give aldehyde derivatives or keto moieties, and these are subsequently oxidized into aldehyde derivatives such as DP728.

## 3. Materials and Methods

### 3.1. Materials

All chemicals used in this work, except where noted, were of analytical grade. Highly purified IOP was obtained from Shanghai Zehan Biopharma Technology Co., Ltd., (Shanghai, China) Mackinawite (60–70% theoretical purity, particle size 60–80 mesh, surface area 15.13 m^2^ g^−1^) was obtained from Shandong West Asia Chemical Industry Co., Ltd., (Shangdong, China) Fe_2_O_3_ (99.5%, 1 μm) was obtained from Shanghai Macklin Biochemical Co., Ltd., (Shanghai, China) Sodium sulfite anhydrous (≥97%), methanol (MeOH, ≥99.5%), ethanol (EtOH, ≥99.7%), sulfuric acid (95%–98%), and sodium hydroxide (≥96%) were purchased from Sinopharm Chemical Reagent Co., Ltd., (Shanghai, China) *tert*-Butyl alcohol (TBA≥99%) was from Aladdin Industrial Corporation. Ultrapure water (18.2 MΩ·cm) was used throughout the study.

### 3.2. Experiment

All batch experiments were carried out in a 550 mL beaker and in an open atmospheric condition. A 500 mL solution with IOP and sulfite at the desired concentration was added into the beaker and constantly stirred at a speed of 400 revolutions per minute with a polytetrafluoroethylene-coated electric stirrer. A predetermined amount of 0.5 g FeS was dosed into the IOP–sulfite solution. The pH value of the solution was adjusted using 0.25 M sodium hydroxide or sulfuric acid as quickly as possible. Each batch of solutions was maintained at a controlled temperature of 25 ± 2 °C during the entire experiment. At specific time intervals, a 2 mL sample was withdrawn from the vessel and then filtered using a polyethersulfone filter (0.45 μm), and then 1.125 mL of the filtrate was placed in a 2 mL vial with 0.375 mL of methanol as the terminating agent before the quantification. The error bars in each figure represent the standard deviation for at least thrice repeated experiments.

### 3.3. Analytical Methods

#### 3.3.1. Characterization

The surface morphologies and chemical compositions of FeS particles were performed using an ultra-high resolution scanning electron microscope (SEM, SU810, Hitachi, Japan) and an energy-dispersive spectrometer (EDS, Bruker xflash 6l60). X-ray diffraction analysis (XRD, D-8, Bruker-axs, Germany) was performed for 2*θ* from 20° to 80°. The FTIR analysis was carried out with Thermo Scientific Nicolet iS20 ranging from 400 to 4000 cm^−1^. 

#### 3.3.2. High-Performance Liquid Chromatography (HPLC)

In this experiment, the IOP concentration was measured via high-performance liquid chromatography (Agilent 1260 Infinity, Agilent chromatography, Perris, CA, USA). The chromatographic separation was performed using a reversed-phase C18 column (250 mm × 4.6 mm, AkzoNobel Kromasil, Bohus, Sweden). The solution of the mobile phase was a mixture of acetonitrile and water at a ratio of 8:92 (*v*/*v*) flowing at 1 mL min^−1^. The wavelength of the UV detector was 238 nm, and the column temperature was maintained at 25 °C.

#### 3.3.3. Liquid Chromatography-MS

The samples used to identify the byproducts of IOP degradation require pretreatment before the quantified analysis. The samples with byproducts were concentrated via solid-phase extraction (SPE) method using an HLB solid-phase extraction column (200 mg sorbent, 6 mL, Jiangsu Green Union Scientific Instrument Co., LTD., Jiangsu, China). A solution containing 6 mL of CH_3_OH with 0.25% (*v*/*v*) formic acid, followed by 5 mL of ultrapure water was used for preliminary cleaning of the cartridge. Samples of 500 mL were percolated at a flow rate of 10 mL min^−1^. A total of 6 mL of 0.25% (*v*/*v*) formic acid in CH_3_OH was added for elution. The eluates were concentrated to 1 mL with a gentle nitrogen flux. The final concentrated samples were analyzed by LC-MS. The chromatographic separations were carried out using an Agilent ZORBAX SB-C18 (150 × 2.1 mm × 3.5 μm particle size) column at a flow rate of 300 μL min^−1^ and monitored using an MS analyzer (6530 Q-TOF, Agilent Technologies, USA). The column temperature was maintained at 30 °C. The solution of the mobile phase was acetonitrile (with 0.05% formic acid, *v*/*v*) when run on ESI positive mode.

#### 3.3.4. The Other Analytical Methods

The total organic carbon (TOC) was measured on an Analytik Jena N/C 300 (Analytik Jena AG, Langewiesen, Germany) TOC analyzer. The pH values were measured via an FE28 pH meter (Mettler Toledo, Switzerland) equipped with a LE438 probe. The concentrations of dissolved oxygen were determined using a JPB-607A dissolved-oxygen analyzer (Xian Yima Optoelec Co., Ltd., Shaanxi, China).

## 4. Conclusions

The FeS/sulfite system exhibits excellent efficiency in the oxidation of IOP, and can achieve over 70% mineralization in 1 h. The optimal dosage of FeS and sulfite are 1 g L^−1^ and 400 μmol L^−1^, at pH = 8. By performing radical quenching experiments, hydroxyl and oxysulfur radicals were identified as the main contributors to the oxidation of IOP. Three primary products were determined, and the possible pathway for IOP degradation, involving H-abstraction and oxidative decarboxylation, was estimated. The results of this study indicate the system’s potential for the degradation and mineralization of IOP. This work is helpful for developing a new approach for the removal of ICM-containing water and extending the applications of SR-AOPs on the water treatment field.

## Data Availability

Data are contained within the article.

## Supplementary Materials

The following are available online, Figure S1: (a) The XRD pattern of raw FeS; (b) EDS images and chemical compositions on the surface of raw FeS; (c) FTIR spectra of raw and residual FeS; Figure S2: Species distribution of 0.01 mM Fe(II) in aqueous solutions at pH in the range of 3–12, Figure S3: Species distribution of 0.4 mM S(IV) in aqueous solutions at pH in the range of 1–12, Figure S4: Changes of pH at different intial pH values, Figure S5: Effects of initial sulfite concentrations on the degradation of IOP by the FeS/sulfite system on IOP degradation, Figure S6: (a) Effects of Cl^−^ on the degradation of IOP. (b) Effects of NO_3_^−^ on the degradation of IOP. (c) Effects of CO_3_^2−^ on the degradation of IOP, Figure S7: (a) Mass spectrum of DP789, (b) mass spectrum of TPD60 and (c) mass spectrum of DP728, Table S1. Mass spectra results for and its degradation products

## Sample Availability

The materials used in the study are commercially available and can be purchased from the relevant firms.
