# Supplementary material for: Efficient Degradation of Iopromide by Using Sulfite Activated with Mackinawite
Source: Molecules. 2021 Oct 28;26(21):6527. doi: 10.3390/molecules26216527 (PMC8588147; doi:10.3390/molecules26216527)
Supplement: Supplementary file 1 [file molecules-26-06527-s001.zip › molecules-1394801-supplementary.pdf]

Supplementary Material

for

Efficient degradation of Iopromide by using sulfite activated with mackinawite

Lists of captions:

Figure S1 (a) The XRD pattern of raw FeS; (b) EDS images and chemical compositions on thesurface of raw FeS;(c) FTIR spectra of raw and residual FeS. 3

Figure S2. Species distribution of 0.01 mM Fe(II) in aqueous solutions at pH in the range of 3 – 12. 4

Figure S3. Species distribution of 0.4 mM S(IV) in aqueous solutions at pH in the range of 1 – 12. 4

Figure S4. Changes of pH at different intial pH values..6Figure S5. Effects of initial sulfite concentrations on the degradation of IOP by the FeS/sulfite system on IOP degradation. 5

Figure S6. (a) Effects of Cl- on the degradation of IOP. (b) Effects of NO3- on the degradation of IOP. (c) Effects of CO32- on the degradation of IOP. 6

Figure S7 (a) Mass spectrum of DP789, (b) Mass spectrum of TPD60 and (c) Mass spectrum of DP728. 8

Table S1. Mass spectra results for and its degradation products 10

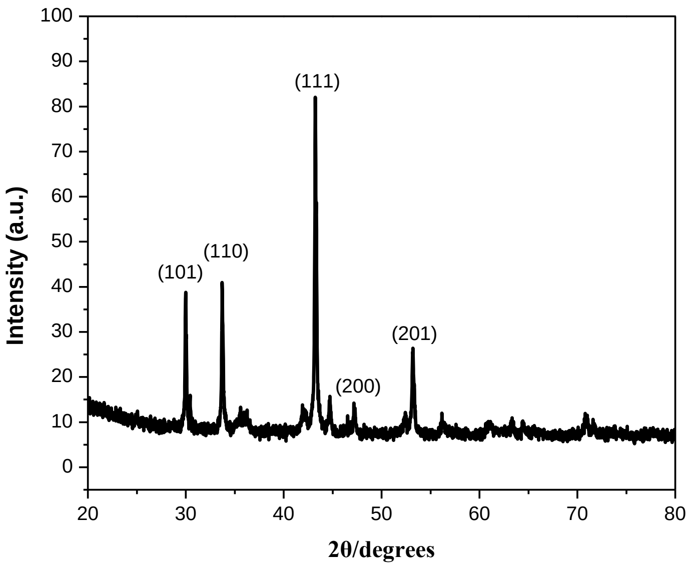

(a)

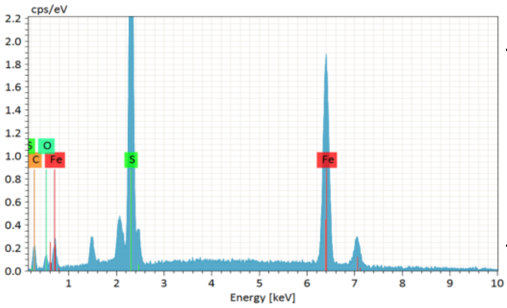

Element	Weight%	Atomic%
Fe	60.85	42.95
S	34.52	42.43
C	3.93	12.89
O	0.7	1.73
Totals	100	100

(b)

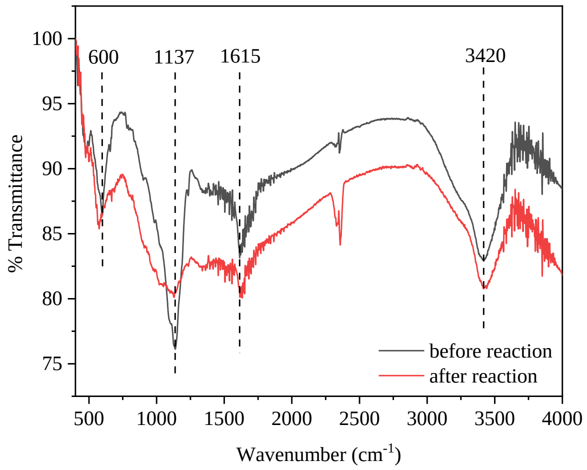

(c)

Figure S1. (a) The XRD pattern of raw FeS; (b) EDS images and chemical compositions on thesurface of raw FeS;(c) FTIR spectra of raw and residual FeS.

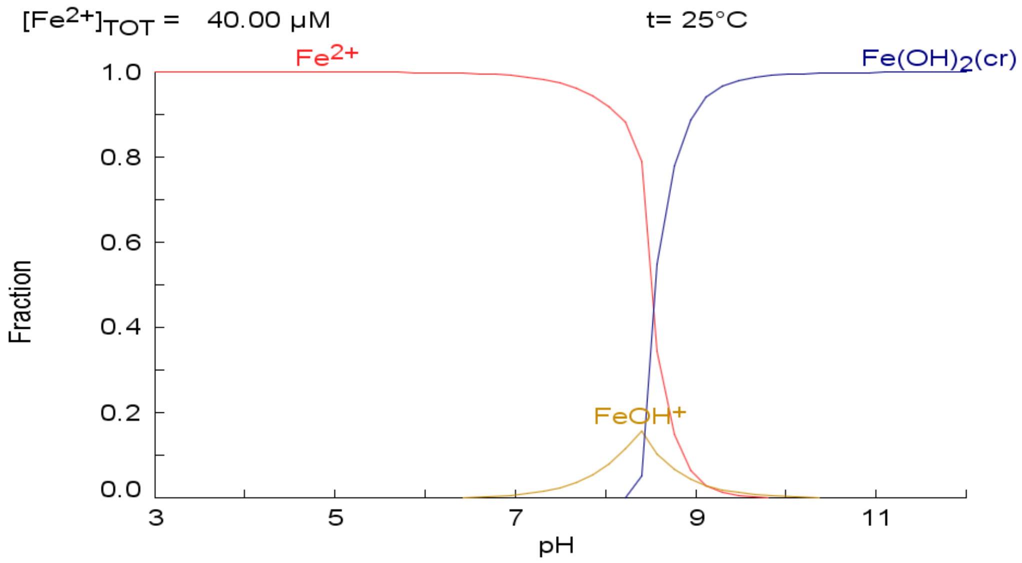

Figure S2. Species distribution of 0.01 mM Fe(II) in aqueous solutions at pH in the range of 3–12.

This figure was created by the special software MEDUSA, which can be downloaded from https://sites.google.com/site/chemdiagr/

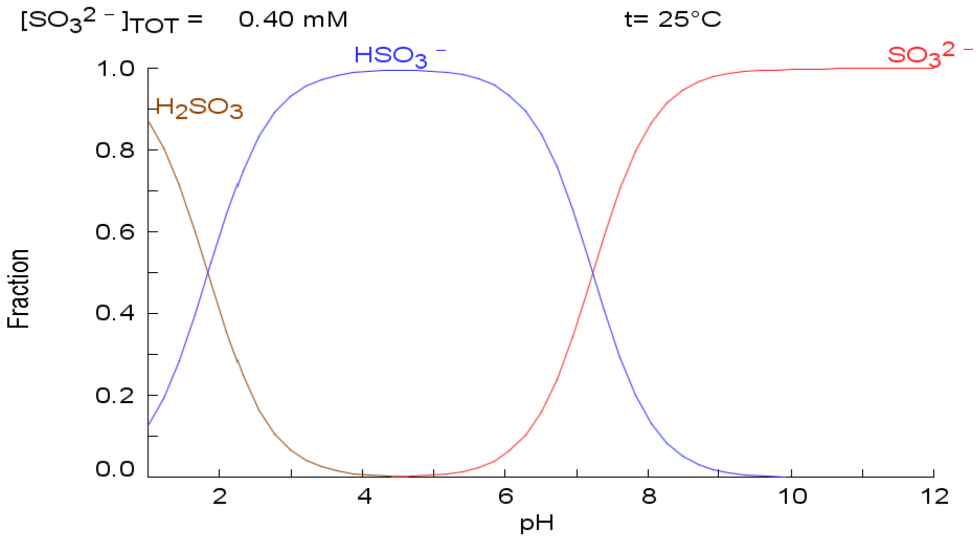

Figure S3. Species distribution of 0.4 mM S(IV) in aqueous solutions at pH in the range of 1–12.

This figure was created by the special software MEDUSA, which can be downloaded from https://sites.google.com/site/chemdiagr/.

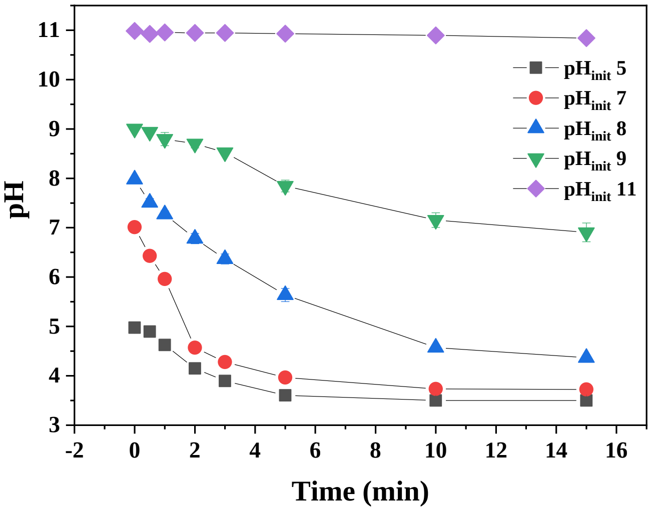

Figure S4. Changes of pH at different initial pH values.

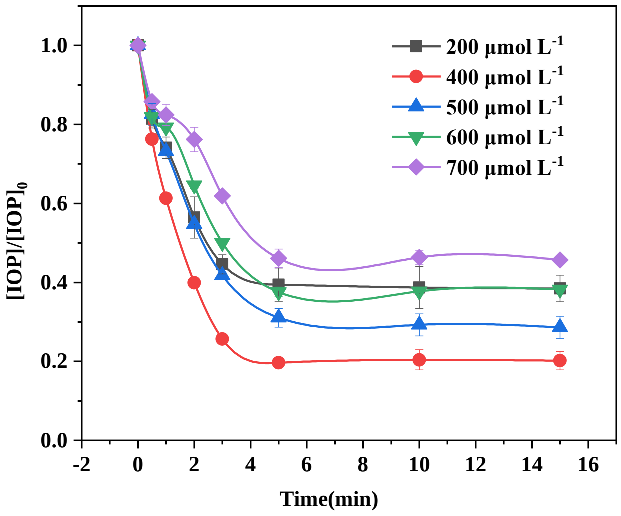

Figure S5. Effects of initial sulfite concentrations on the degradation of IOP by the FeS/sulfite system on IOP degradation.

Conditions: $[IOP]_0$ = 1.5 mg L^{-1}, $[FeS]_0$ = 1 g L^{-1}, $[Na_2SO_3]_0$ =200-700 μmol L^{-1}, pH_{init} = 8.0.

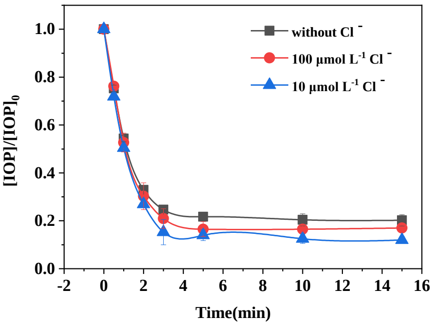

(a)

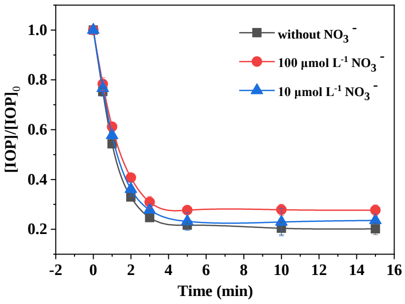

(b)

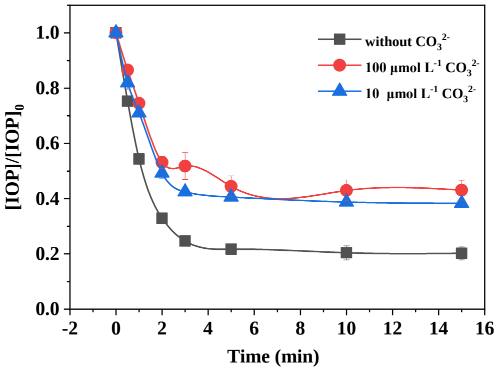

(c)

Figure S6. (a) Effects of Cl^- on the degradation of IOP. **(b)** Effects of NO_3^- on the degradation of IOP. **(c)** Effects of CO_3^{2-} on the degradation of IOP.

Conditions: $[IOP]_0$ = 1.5 mg L^{-1}; $[FeS]_0$ = 1 g L^{-1}; $[Na_2SO_3]_0$ = 400 μmol L^{-1}; pH_{init} = 8.0.

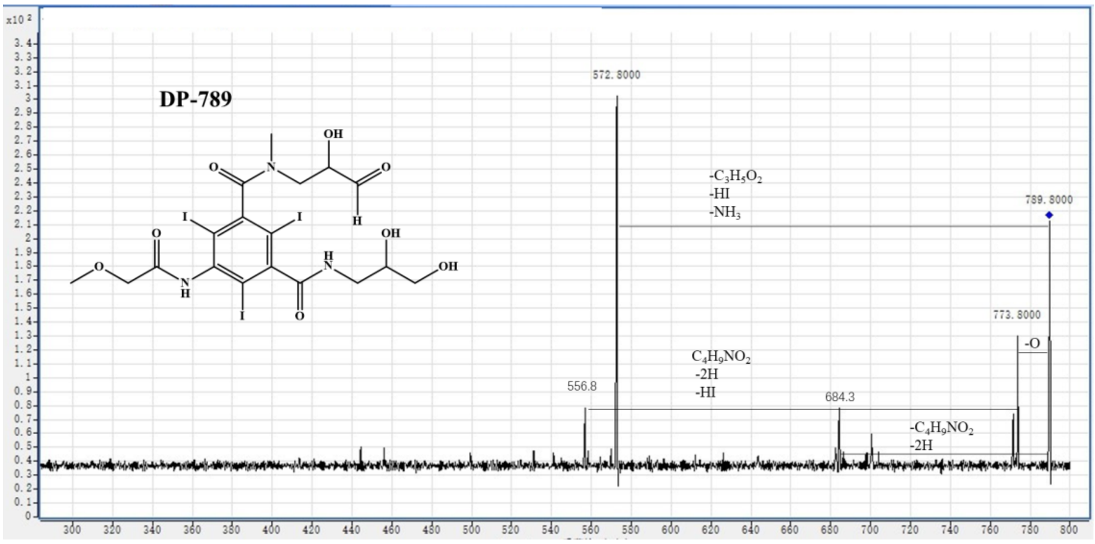

(a)

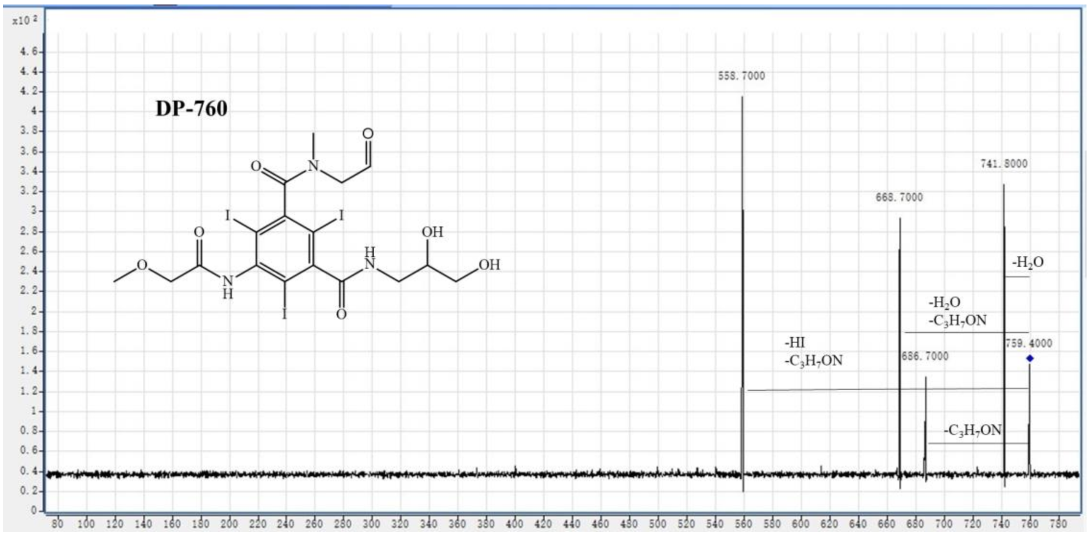

(b)

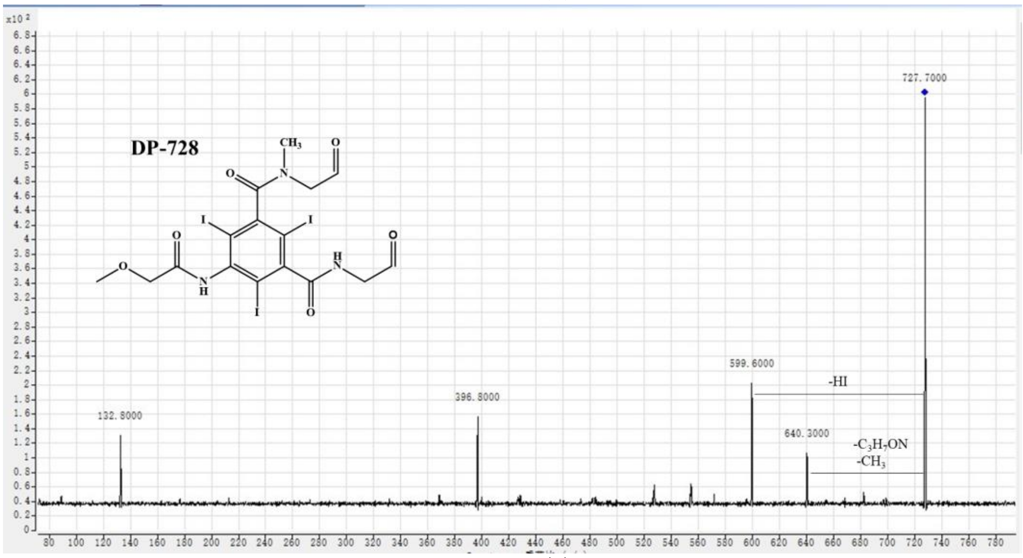

(c)

Figure S7 (a) Mass spectrum of DP789, **(b)** Mass spectrum of TPD60 and **(c)** Mass spectrum of DP728.

Table S1. Mass spectra results for and its degradation products.

Name	Ion[M + H]$^+$	Elemental composition	Losses to give fragments	Structure proposed
IOP	791.8	$C_{18}H_{24}I_3N_3O_8$		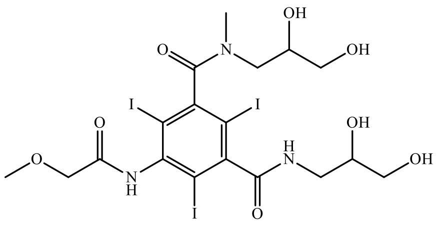
DP789	789.8<br>773.8<br>684.3<br>556.8<br>572.8	$C_{18}H_{22}I_3N_3O_8$	-2H<br>-O<br>-$C_4H_9NO_2$; -2H<br>-$C_4H_9NO_2$; -2H ;-HI<br>-$C_3H_5O_2$; -HI;-NH_3	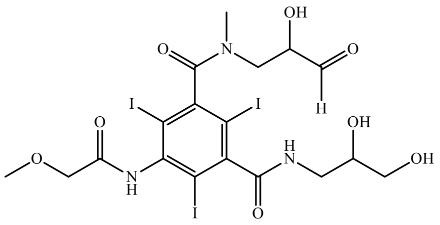
DP760	759.8<br>741.8<br>686.7<br>668.7<br>558.7	$C_{17}H_{20}$ $I_3N_3O_7$	-2H; -CHOH<br>-H_2O<br>-C_3H_7ON<br>-C_3H_7ON; -H_2O<br>-C_3H_7ON; -HI	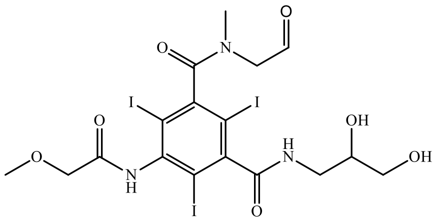
DP728	727.8<br>599.6<br>640.3	$C_{16}H_{16}I_3N_3O_6$	-2H; -CH_2O;-CH_3OH<br>-HI<br>-C_3H_7ON; -CH_3	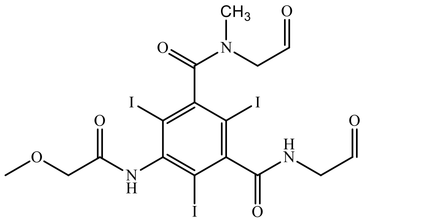
